# Risk for Seasonal Affective Disorder (SAD) Linked to Circadian Clock Gene Variants

**DOI:** 10.3390/biology12121532

**Published:** 2023-12-15

**Authors:** Thanh Dang, William A. Russel, Tazmilur Saad, Luvna Dhawka, Ahmet Ay, Krista K. Ingram

**Affiliations:** 1 Department of Computer Science, Colgate University, Hamilton, NY 13346, USA; thdang@colgate.edu (T.D.); ssaad@colgate.edu (T.S.); 2 Department of Biology, Colgate University, Hamilton, NY 13346, USA; wrussel@colgate.edu (W.A.R.); aay@colgate.edu (A.A.); 3 Department of Mathematics, Colgate University, Hamilton, NY 13346, USA; 4 Feil Family Brain & Mind Research Institute, Weill Cornell Medicine, New York, NY 10065, USA

**Keywords:** circadian clock, seasonal affective disorder, SAD, depression, seasonality, machine learning, mood disorders, chronobiology, molecular clockwork

## Abstract

**Simple Summary:**

Depression and related mood disorders, such as seasonal affective disorder (SAD), affect nearly one-fifth of the global population and have substantial economic and personal costs. Mutations in circadian clock genes are associated with mood disorders, suggesting that molecular pathways affecting mood are influenced by the circadian clock, but the molecular mechanisms underlying this link are poorly understood. We use machine learning and statistical analyses to determine the circadian gene variants and clinical features most highly associated with symptoms of seasonality and SAD. Our results support previous links between CRY2, PER2, and ZBTB20 genes and identify novel links for CLOCK and PER3 with symptoms of seasonality and SAD. Our findings reinforce the sex-specific influences of circadian genes on seasonality and SAD and highlight the multiple pathways by which clock variants affect downstream mood pathways via direct and indirect mechanisms. These results enhance our understanding of the molecular mechanisms underlying seasonality in mood and may elucidate new therapeutic targets for circadian-related mood disorders, including SAD, depression, and anxiety.

**Abstract:**

Molecular pathways affecting mood are associated with circadian clock gene variants and are influenced, in part, by the circadian clock, but the molecular mechanisms underlying this link are poorly understood. We use machine learning and statistical analyses to determine the circadian gene variants and clinical features most highly associated with symptoms of seasonality and seasonal affective disorder (SAD) in a deeply phenotyped population sample. We report sex-specific clock gene effects on seasonality and SAD symptoms; genotypic combinations of CLOCK3111/ZBTB20 and PER2/PER3B were significant genetic risk factors for males, and CRY2/PER3C and CRY2/PER3-VNTR were significant risk factors for females. Anxiety, eveningness, and increasing age were significant clinical risk factors for seasonality and SAD for females. Protective factors for SAD symptoms (in females only) included single gene variants: CRY1-GG and PER3-VNTR-4,5. Clock gene effects were partially or fully mediated by diurnal preference or chronotype, suggesting multiple indirect effects of clock genes on seasonality symptoms. Interestingly, protective effects of CRY1-GG, PER3-VNTR-4,5, and ZBTB20 genotypes on seasonality and depression were not mediated by chronotype, suggesting some clock variants have direct effects on depressive symptoms related to SAD. Our results support previous links between CRY2, PER2, and ZBTB20 genes and identify novel links for CLOCK and PER3 with symptoms of seasonality and SAD. Our findings reinforce the sex-specific nature of circadian clock influences on seasonality and SAD and underscore the multiple pathways by which clock variants affect downstream mood pathways via direct and indirect mechanisms.

## 1. Introduction

The global prevalence of depression and related mood disorders, including seasonal affective disorder, incurs significant personal and economic costs [1,2]. Individuals affected by mood disorders are frequently plagued by sleep problems and disruptions in their circadian rhythms and daily cycles in physiological processes and behavior, indicating a connection between circadian physiology and mood pathways [3,4,5]. In addition, mutations in core regulatory clock genes are associated with sleep–wake patterns and mood disorders, suggesting that molecular pathways regulated by the circadian clock may influence mood [6].

Circadian misalignment or ‘social jetlag’, the degree to which the circadian phase of an individual is advanced or delayed relative to their entrained (or socially influenced, i.e., work demands) sleep–wake cycle, is a likely factor influencing negative affect [7,8,9,10,11]. Multiple candidate gene studies have reported circadian clock polymorphisms associated with both chronotype and mood disorders [4,5,12,13,14,15,16,17], with some clock variants displaying sex-specific patterns [18]. Genome-wide association studies (GWAS) also link circadian genes with chronotype, depression, and other psychological disorders [19,20,21,22]. In addition, studies examining individuals with severe circadian disorders, such as the familial advanced sleep phase (FASP) and delayed sleep phase (DSP) phenotypes, support the high frequency of comorbidity between circadian disorders and negative affect [6,22,23]. Although the results from these studies show robust support for the influence of circadian misalignment on mood, the mechanisms driving these gene-environment interactions are poorly understood.

Seasonal affective disorder (SAD) is an excellent model with which to test for gene–environment interactions. SAD is a sub-type of depression that normally occurs in the fall/winter seasons and is most frequently reported at latitudes that experience shorter photoperiods or hours of daylight (but see [24] for a review of the heterogeneity of SAD symptoms in general populations). The disorder is associated with hypersomnia, social withdrawal, overeating, low energy, and carbohydrate cravings [25]. The diagnosis of SAD requires that patients experience symptoms for a minimum of two years. SAD’s prevalence in the general population can be as high as 4–15%, and SAD is four times more prevalent in women [25]. While depression and anxiety can have a myriad of contributing risk factors [26], seasonal affective disorder is likely to be more tightly linked to the circadian clock. Another important consideration is the fact that global populations harbor diverse genetic backgrounds that may or may not be well-adapted to short-light days. There is ample evidence to suggest that frequencies of circadian gene variants differ with latitude, population history, and environmental factors [27]. Finally, the prevalence of SAD tends to be higher in females. A recent study highlighted the sex-specific association of circadian gene variants with MDD [18], suggesting the possibility that sex-specific molecular pathways may be linked to SAD.

Large-scale approaches to genomic association studies (e.g., GWAS) utilize genomic databases to identify single gene variants associated with a phenotype, including complex mental health disorders like seasonal depression. Still, many GWAS studies fail to identify clinically or biologically relevant associations. Newly described methods, like phenome-wide association studies (PheWAS), attempt to solve this issue by using a genotype-to-phenotype approach utilizing medical databases with more robust phenotyping. However, these studies are costly, and it is still challenging to extrapolate beyond single-gene associations.

In this study, we seek to explore a complementary approach to determine key genetic associations of genotypes with complex behavioral disorders. Taking advantage of PheWAS approaches, we use a combination of machine learning and statistical approaches to identify haplotypes or combinations of multi-gene variants associated with SAD in a population of individuals that are deeply phenotyped. This approach is particularly relevant for the study of complex behavioral disorders, as previous studies of genetic influence on mental health strongly suggest an increased risk due to the additive effects of multiple gene variants [4,17,28]. It is also becoming increasingly clear that environmentally influenced mental health disorders, like seasonal affective disorder, are strongly influenced by genes–environment interactions. These subtleties are difficult to test using ‘lightly’ phenotyped or non-clinical population samples.

In cross-sectional studies, we and others have found significant associations between circadian gene variants and depression, anxiety, sleep disturbance, sleep architecture, and SAD (reviewed in [29,30]). Our longitudinal study in a predominantly Caucasian population found a prevalence of 12.5% for SAD symptoms, with the risk of SAD being five times higher in females than in males. By targeting previously identified SNPs from depressive mood disorders, we use a deeply phenotyped population sample to identify sex-specific genetic associations of SAD with circadian gene variants, clinical features (depressive symptoms/anxiety symptoms/chronotype/sleep issues, gender), and environmental features (seasons, socioeconomic status) using machine learning and Bayesian approaches.

## 2. Materials and Methods

The genetic and statistical methods described below were used in our previous study on circadian influences on anxiety with slight modifications in this study. More details can be found in Zafar et al., 2022 [31].

### 2.1. Participants

Data were collected from a multi-year study of a population of predominantly young Caucasian adults (total sample: *n* = 891; participants with complete genetic data: *n* = 273; 76 males, 197 females; median age: 19, range 17–79). Twenty-nine participants were faculty at the university; removing these older individuals did not alter the results, so they were included in the study. Participants were recruited primarily via an introductory Biology course comprising first- or second-year undergraduates during the years 2013–2019. Sampling occurred in late fall or early spring (November–March). The participation rate for each sampling period ranged from 94 to 99%. All methods adhered to the principles of the Declaration of Helsinki; the Institutional Review Board at Colgate University approved all procedures and consent forms (#FR-F13-07, #ER-F14-12, #F15-13, #ER-F16-19). All participants gave written informed consent.

### 2.2. Self-Report Surveys

We established a dataset using deep phenotyping via computer-based surveys, including the Seasonal Pattern Assessment Questionnaire (SPAQ (*n* = 221); Rosenthal et al., 1984 [32]), the Beck Depression Inventory (BDI-II (*n* = 254); Beck and Beamesderfer 1974 [33]), Spielberger’s State–Trait Anxiety Scale (State–Trait Anxiety Inventory (STAI; Spielberger et al., 1983 [34]), and the short form of the Patient-Reported Outcomes Measurement Information System Sleep Disturbance (PROMIS^®^; Yu et al., 2012 [35]). SPAQ is a self-reported measure of seasonality and seasonal affective disorder, ranging from 0 to 24, with scores < 11 indicating non-seasonality. BDI-II is a diagnosis of depression, combining scores on screening tests with clinical interviews. BDI-II scores are treated as quantized values and range from 0 to 60; individuals with scores < 14 are not depressed, those with 14–19 are mildly depressed, those with 20–28 are moderately depressed, and those with >28 are severely depressed. SAD is a diagnostic measure for seasonal depression, already recorded in binary. The Munich Chronotype Questionnaire (MCTQ; Roennenberg et al., 2007 [36]) is a self-reported measure of sleep–wake chronotype; mid-sleep point on a free day was used to designate the chronotype score, with the highest quartile MSF scores indicating eveningness (F:M ratio = 2.5) and the lowest quartile scores indicating morningness (F:M ratio = 5.8). Social jetlag was measured as MSF–MSW, the mid-sleep on workdays. We also reported diurnal preference using the Morningness–Eveningness Questionnaire (MEQ; Horne and Ostberg, 1976 [37]).

### 2.3. Genotyping

DNA was extracted from 10 to 20 hair follicles from each participant. Hair follicles were digested for 24 h at 56 °C with Proteinase K, and these samples were further purified using the Qiagen DNAeasy Micro Kit. Using TaqMan SNP Genotyping assays (Applied Biosystems, Foster City, CA, USA), we identified single-nucleotide polymorphisms (SNPs) using real-time qPCR protocols on an ABI 3700HT instrument. Homozygous or heterozygous genotypes for the major and minor alleles were recorded for each participant.

The PER3-VNTR length polymorphism repeat region was analyzed via fragment length analysis on an ABI 3100 sequencer using GeneScan^®^ software (1.0). We fluorescently labeled the forward primer with 6-FAM (5′-CAAAATTTTATGACACTACCAGAAT GGCTGAC-3′). PCR was performed using the forward and reverse primer (5′-AACC TTGTACTTCCACATCAGTGCCTGG-3′) The 25-μL reactions were run with the following cycling conditions: 3 min at 94 °C, 35 cycles of 45 s at 94 °C, 45 s at 58 °C, and 45 s at 72 °C, with a final 3 min step at 72 °C. PER3 alleles were separated by repeat number/size as PER3 4/4, PER3 4/5, or PER3 5/5 and recorded for each participant.

### 2.4. Feature Generation, Selection and Classification

Each individual has nine genotypic features—*CLOCK3111* (rs1801260), *CRY1* (rs2287161), *CRY2* (rs10838524), *PER2* (rs10462023), *PER3A* (rs228697), *PER3B* (rs17031614), *PER3C* (rs10462020), *PER3VNTR* (rs57875989), and *ZBTB20* (rs139459337)—which can be homozygous dominant, homozygous recessive, or heterozygous. We also collect five behavioral/clinical features: gender, diurnal preference scores (MEQ and MSF), age (≤22 or >22), and socioeconomic status (poor, lower-middle-class, upper-middle-class, affluent). We thus generated 14 features (we will refer to these as one-way features). We further created pairwise interactions among the nine genotypic features and their respective variants, generating 9C2*9 additional features (we will refer to these as two-way features).

We performed one-hot-encoding for the non-binary categorical features. To reduce multicollinearity and high dimensionality, we further removed the most frequent group for each feature as the reference point, generating a total of 286 features in our expanded dataset. The final dataset for each disorder target is obtained by stratifying the expanded data by the respective disorder metric (SPAQ, SAD, or BDI) and removing encoded features that were not present in any sample. The corresponding dimensions of these final datasets are as follows: 221 subjects and 261 features for SPAQ, 152 subjects and 258 features for SAD, and 254 subjects and 268 features for BDI, respectively.

Datasets are cleaned, fully imputed with k-nearest neighbors’ imputation [38], and normalized before the machine learning process. When the dataset is imbalanced, the synthetic minority oversampling technique (SMOTE) is employed with k = 5 to ensure equal numbers of samples for positive and negative cases. Specifically, SMOTE creates an artificial sample by randomly selecting a point between a minority class sample and another random neighbor in its k-neighbor feature space [39].

We analyzed the dataset through a ten-fold cross-validation process. In this process, the data are split into 10 subsets. Each subset is used once as a test set, while the remaining ninety percent of the data serve as the training set. Stratified sampling was used to split the training and testing sets to avoid overfitting or underfitting. This cross-validation procedure was repeated ten times. During each iteration, we aggregated the features selected in each fold, applying this across all the methods we used. We repeated the procedure described below ten times and aggregated the features selected at each of the folds across all listed methods. We used five feature selection methods to find features such as epistatic combinations that are possible robust predictors of mood disorders. Two ranking-based feature selection methods, Chi-squared (CHI2) and ReliefF (ReF), sort features in order of correlation with the disorder [40,41]. Three subset-based feature selection methods—correlation-based feature selection (CFS), maximum relevance minimum redundancy (MRMR), and joint mutual information (JMI)—use information theory-based criteria to identify potential subsets from the feature space [42,43,44]. Each feature selection method was run on the expanded datasets using ten-fold cross-validation. We then extracted copies of the dataset which included only the top-20 most frequently selected features.

We applied four classifiers, including logistic regression (LR), random forests (RF), support vector machine (SVM), and XGBoost (XGB), on the features selected for the training subsets [45,46,47]. Grid search is used to identify the combination of hyperparameters that yields the model with the highest accuracy. This final model is applied to the test data to predict the labels, and the results are evaluated accordingly based on model accuracy.

### 2.5. Statistical Analyses

We ran all of the following analyses separately, with each of SPAQ, SAD, and BDI being the target variable with R [48]. Each analysis (except for ANOVA and ARACNE) was run three times each for all participants and each gender separately. We also used the top-20 datasets wherever possible to see if results from a more-robust dataset match the overall trends.

We ran univariate logistic regression on features selected in 80% of the runs by at least three out of the five feature selection methods to determine directionality, as they are likely effective predictors on their own. After this univariate analysis, we performed multivariate logistic regression on the top-20 risk factors to see which factors were significant predictors of mood disorders when used in tandem with other features. We recorded their odds ratios, *p*-values and 95% confidence intervals. Multivariate linear regression was used for SPAQ and BDI metrics but not SAD since the target variable is categorical. Due to the high-dimensional configuration, we performed multivariate logistic and linear regression with stepwise selection using Akaike Information Criterion (AIC) to create an optimal model without compromising the model accuracy [49].

We used the *mediation* package in R to determine the mediation effects of diurnal preference (MEQ and MSF) in the relationship between independent variables (clinical/behavioral factors) and the dependent variable (mood disorder). We fit the mediator and outcome models with linear regression and used the *mediate* function with non-parametric bootstrap for variance estimation [50]. The results are summarized numerically with point estimates, confidence intervals, and the *p*-values for the average direct, indirect, and total effects.

For the SPAQ and BDI target variables, the subset of genotypes determined significant via univariate and multivariate regression were analyzed in-depth with type-III sums of squares two-way ANOVA. Gender was the second independent variable as we targeted sex-specific paths [51].

For each gender, using one-way features, we performed association analysis to identify likely relations based on measures of interest. We transformed all the variables into categorical data and applied the a priori algorithm in the *arules* package in R [52] to obtain rules of the form X → Y. X refers to the multi-way genotype and clinical feature combination’s increasing risks of acquiring Y, the mood disorder. We set the baseline threshold of rule size to, at most, 6, with minimum support of 0.04 and confidence of 0.7, and then we sorted the rules by their respective lift values. Network plots were created using the *igraph* library [53], with node size representing support and edge width representing confidence.

We used the algorithm for the reconstruction of gene regulatory networks (ARACNE) [54] to identify the direct and indirect relationships between one-way features and the disorder target via diurnal preference scores (MEQ and MSF). We employed the *mi.empirical* method for ARACNE to compute distance metrics. We constructed a gene network via the *minet* package as ARACNE analyses each qualified the gene triplet and removes the edge with the smallest metric; then, we plotted it using Rgraphviz v2.46.0 [55,56]. ARACNE calculates the frequency of a link via bootstrapping, and we kept strong links by setting a threshold of 46% for bootstrap support. The dataset and code are available at https://github.com/thanhdang2712/Mood-Disorder-Project.

## 3. Results

### 3.1. Seasonality Prediction

**Figure 1:**
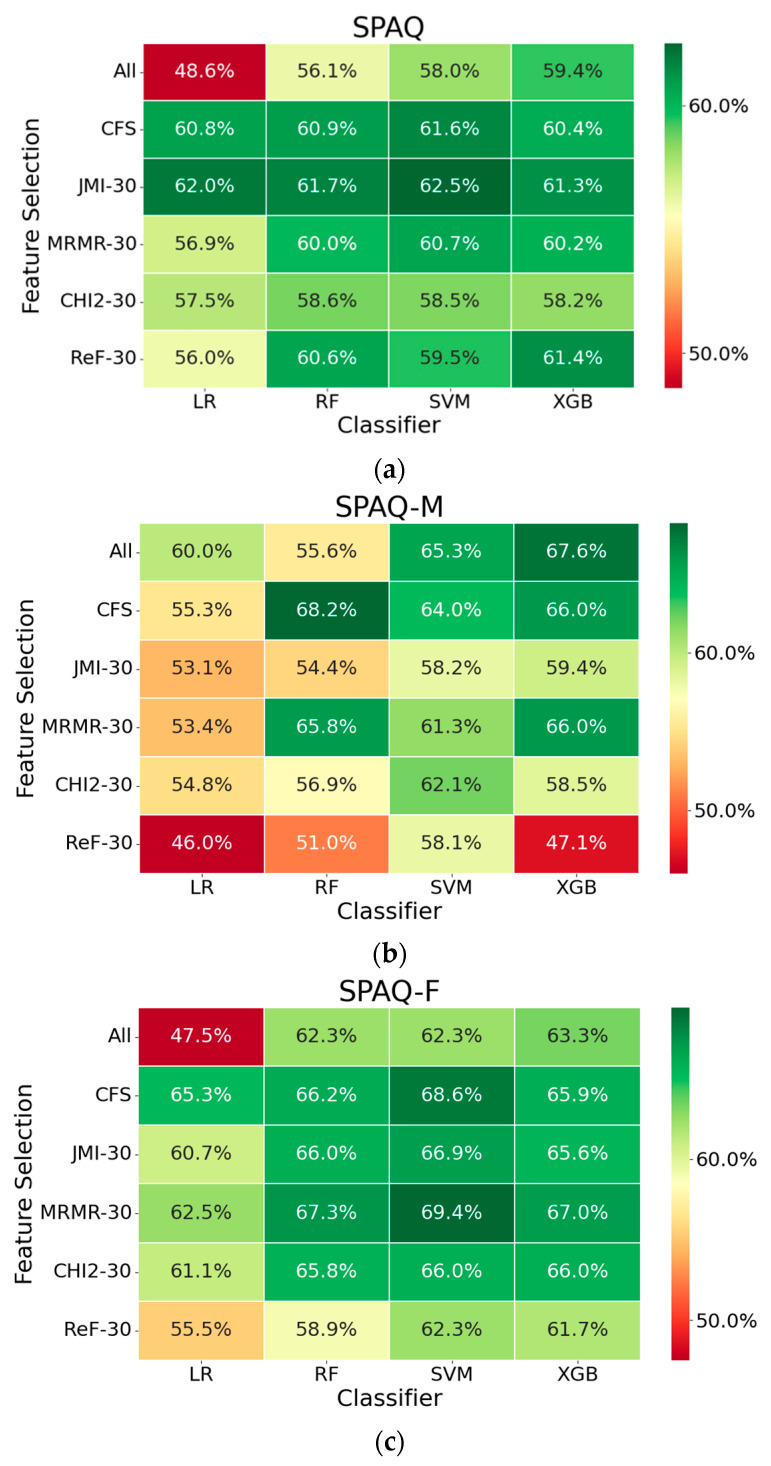
Classifiers exhibit greater accuracy increase from baseline when stratified by sex. (**a**) Analyses on the overall dataset yielded up to 6% greater accuracy than the baseline accuracy (56.5%) using the support vector machine (SVM) classifier and the joint mutual information (JMI) feature selection. (**b**) Prediction of male seasonality yielded up to 10% greater accuracy than the baseline accuracy (58%) using the RF classifier and correlation-based feature selection. (**c**) Prediction of female seasonality yielded up to 7% greater accuracy than the baseline accuracy (62.2%) using the LR and SVM classifiers and mRMR feature selection.

Seasonality was predicted with 62.5% accuracy, 6% higher than a baseline accuracy of 56.5% (Figure 1a). Sex-stratified classifiers predicted seasonality for males (68.2%) and females (69.4%), with respective ~7% and ~10% increases from baseline (Figure 1b,c).

### 3.2. Sex-Specific Risk and Protective Factors for Seasonality and SAD Symptoms

**Figure 2:**
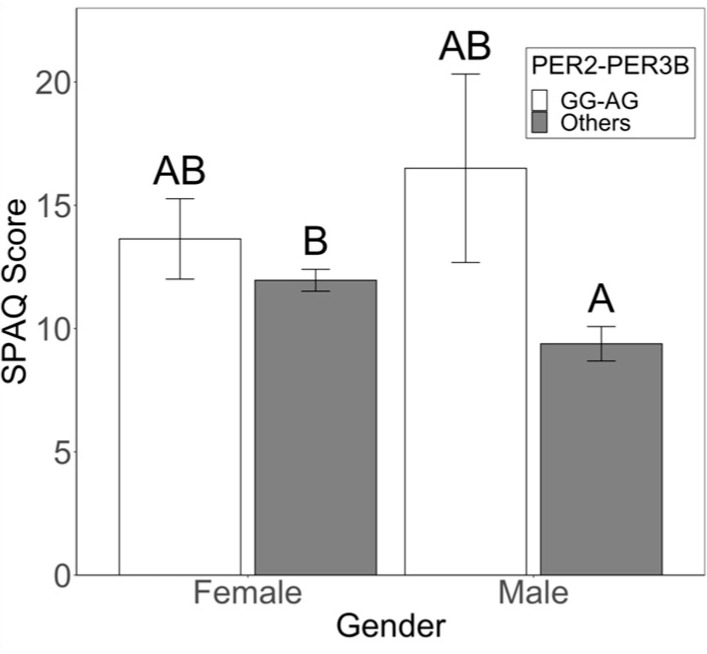
Two-way ANOVA and Tukey post hoc analysis reveal that the PER2-GG/PER3B-AG combination is predictive of seasonality in males. Average SPAQ scores were higher in males with the PER2-GG/PER3B-AG genotypic combination than in males with other genotypic combinations (Genotype: F_1,217_ = 4.312, *p* = 0.039; Gender: F_1,217_ = 0.005, *p* = 0.946; Genotype × Gender: F_1,217_ = 1.650, *p* = 0.200). Error bars denote SE. A, B denote significant differences for Tukey post hoc analyses.

In males, the PER2-GG/PER3B-AG combination was associated with a seven-fold increase in odds of seasonality (Table 1; OR 7.3 (0.87–13.7) adj. *p* = 0.034); SPAQ scores were higher in these males than males with other genotypes (Figure 2; Genotype: F_1,217_ = 4.312, *p* = 0.039; Gender: F_1,217_ = 0.005, *p* = 0.946; Genotype × Gender: F_1,217_ = 1.650, *p* = 0.200). In addition, the CLOCK3111-TC/ZBTB20-AT genotypic combination increased odds of seasonality in males almost four-fold (Table 1; OR 4.0 (0.37–7.58) adj. *p* = 0.052).

**Figure 3:**
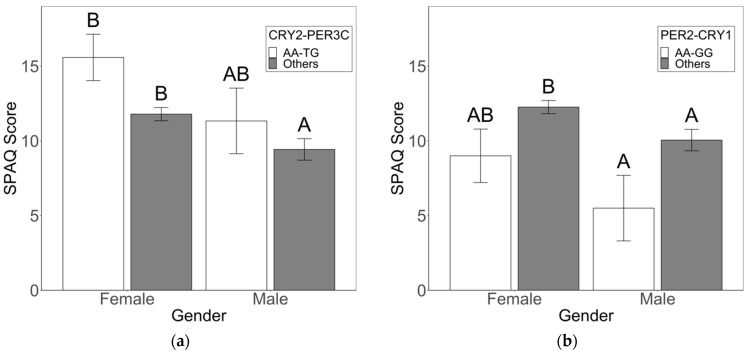

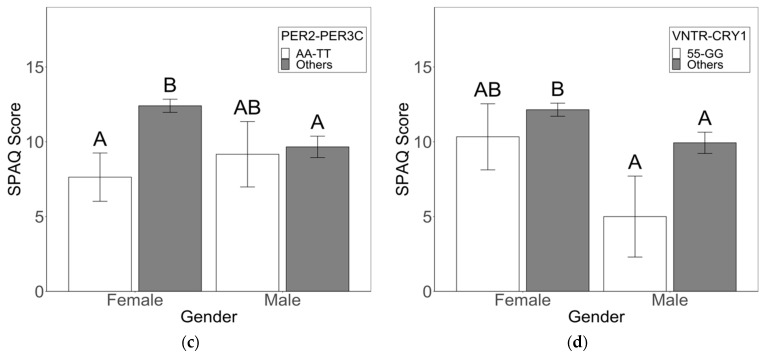
Two-way ANOVA and Tukey post hoc analysis reveal risk and protective combinations for seasonality in females. (**a**) Significantly higher average SPAQ scores were identified in females with the CRY2-AA/PER3C-TG combination relative to other genotypic combinations (Genotype: F_1,217_ = 4.084, *p* = 0.044; Gender: F_1,217_ = 5.496, *p* = 0.020; Genotype × Gender: F_1,217_ = 0.449, *p* = 0.504). (**b**) Average SPAQ scores were lower for males and females with the PER2-AA/CRY1-GG combination (Genotype: F_1,217_ = 7.001, *p* = 0.009; Gender: F_1,217_ = 3.735, *p* = 0.055; Genotype × Gender: F_1,217_ = 0.192, *p* = 0.662). (**c**) Average SPAQ scores were lower for females with the PER2-AA/PER3C-TT combination than for females with other combinations (Genotype: F_1,217_ = 3.420; *p* = 0.066, Gender: F_1,217_ = 0.182; *p* = 0.670, Genotype × Gender: F_1,217_ = 2.257, *p* = 0.134). (**d**) Average SPAQ scores were lower for males and females with VNTR-5,5/CRY1-GG combination (Genotype: F_1,217_ = 3.525, *p* = 0.062; Gender: F_1,217_ = 4.417, *p* = 0.037; Genotype x Gender: F_1,217_ = 0.755, *p* = 0.396) (N = 221). A, B denote significant differences for Tukey post hoc analyses.

In females, the CRY2-AA/PER3-VNTR-4,5 combination was associated with a nine-fold increase in seasonality risk in our logistic regression (Table 1; OR 8.9 (1.71–164.19) adj. *p* = 0.037). Additionally, linear regression identified CRY2-AA/PER3C-TG as a risk factor for seasonality (Table 1; estimate 4.3 (1.55–7.03) adj. *p* = 0.0091), and SPAQ scores were higher in these females than females with other combinations (Figure 3a; Genotype: F_1,217_ = 4.084, *p* = 0.044; Gender: F_1,217_ = 5.496, *p* = 0.020; Genotype × Gender: F_1,217_ = 0.449, *p* = 0.504). Older age and evening-type behavior were clinical risk factors for seasonality in females (Table 1; OR 6.3 (1.49–44.04) adj. *p* = 0.023; OR 1.8 (1.32–2.50) adj. *p* = 0.023).

Logistic regression revealed that the CRY1-GG/PER3-VNTR-5,5 combination was associated with a near ten-fold decrease in the odds of seasonality in females (Table 1; OR 0.1 (0.0058–0.72) adj. *p* = 0.048); males and females with this combination had lower SPAQ scores than individuals with other combinations (Figure 3d; Genotype: F_1,217_ = 3.525, *p* = 0.062; Gender: F_1,217_ = 4.417, *p* = 0.037; Genotype × Gender: F_1,217_ = 0.755, *p* = 0.396). PER2-AA/PER3C-TT reduced the odds of seasonality five-fold (Table 1; OR 0.2 (0.043–0.74) adj. *p* = 0.023); SPAQ scores were lower in females with this genotype combination (Figure 3c; Genotype: F_1,217_ = 3.420, *p* = 0.066; Gender: F_1,217_ = 0.182, *p* = 0.670; Genotype × Gender: F_1,217_ = 2.257, *p* = 0.134). CRY1-GG/PER2-AA was a significant protective factor identified via multivariate linear regression (Table 1; estimate –3.6 (–6.74–−0.45) adj. *p* = 0.050); average SPAQ scores were lower for males and females with this combination relative to other combinations (Figure 3b; Genotype: F_1,217_ = 7.001, *p* = 0.009; Gender: F_1,217_ = 3.735, *p* = 0.055; Genotype × Gender: F_1,217_ = 0.192, *p* = 0.662).

### 3.3. Association Rule Learning Networks Also Reveal Sex-Specific Rules for Seasonality

**Figure 4:**
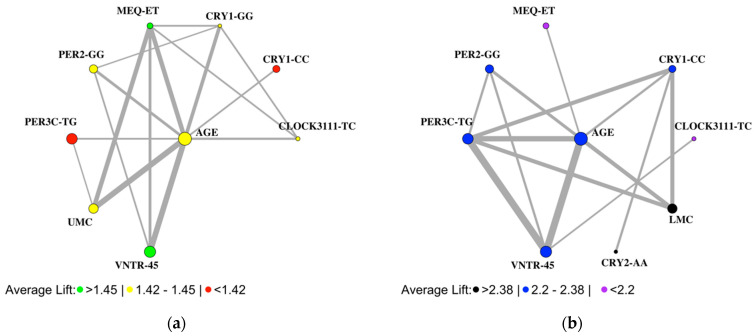
Association rule networks for male and female seasonality. (**a**) The most frequently occurring SNP variants for males included VNTR-4,5, PER3C-TG, PER2-GG, and CRY1-CC, while age and being lower-middle class (LMC) appeared as important clinical factors. (**b**) The most frequently occurring SNP variants for females were VNTR-4,5, PER3C-TG, PER2-GG, and CRY1-CC, while age, being upper-middle class (UMC), and eveningness commonly appeared as important clinical factors.

Association rule learning networks were used to identify co-occurring features that strongly predicted seasonality (Figure 4). In males and females, the most frequently occurring SNP variants associated with seasonality symptoms were CRY1-CC, PER2-GG, PER3C-TG, and PER3-VNTR-4,5 (Figure 4a,b). CRY2-AA and CRY1-GG were SNP variants exclusive to males and females, respectively. PER3C-TG, PER3-VNTR-4,5, and age were the most frequently occurring factors for males, while PER3C-TG, PER3-VNTR, age, being upper middle class, and eveningness were the most frequently occurring factors for females.

**Figure 5:**
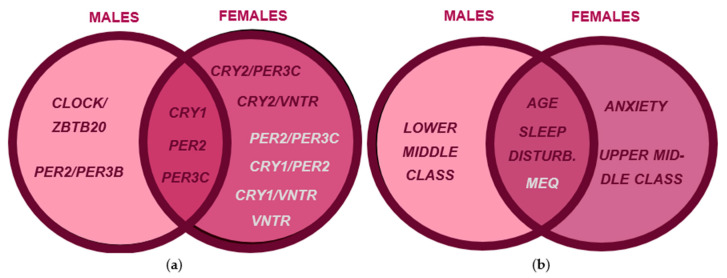
Venn diagram summarizing significant genotypic and clinical features for seasonality in males and females resulting from multivariate linear regression, univariate logistic regression, multivariate logistic regression, and association rule learning network results. Venn diagram comparisons were constructed from male and female seasonality analysis, risk factors were denoted by red text, and protective factors were denoted by silver text. (**a**) Genotypic factor comparison reveals shared and exclusive risk factors between males and females. (**b**) Clinical factor comparison reveals shared and exclusive risk factors between males and females.

We constructed Venn diagrams to summarize sex-specific gene and clinical feature associations with seasonality symptoms (Figure 5). The CLOCK3111-TC/ZBTB20-AT and PER2-GG/PER3B-AG combinations were significant risk factors exclusive to males, while CRY2-AA/PER3C-TG and CRY2-AA/PER3-VNTR-4,5 were significant risk factors exclusive to females. Risk factors for both sexes included CRY1-CC, PER2-GG, PER3C-TG, and PER3-VNTR-4,5 (Figure 5a). Additionally, anxiety and belonging to the upper middle class were risk factors in males, while belonging to the lower middle class was a risk factor for females (Figure 4b). In both sexes, age and sleep disturbance were clinical risk factors, and diurnal preference was protective (Figure 5b).

### 3.4. Comparisons of Genotypic and Clinical Feature Associations with Seasonality and SAD

**Figure 6:**
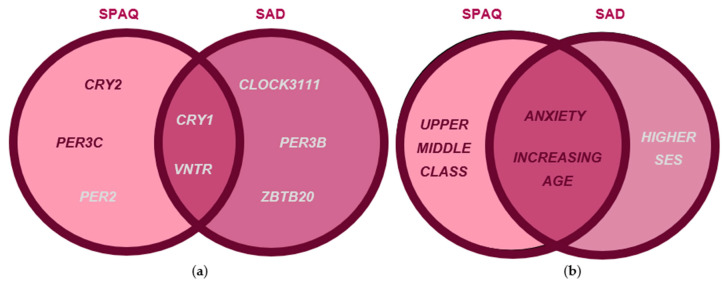
Venn diagram summarizing significant genotypic features for female seasonality and female SAD resulting from multivariate linear regression, univariate logistic regression, multivariate logistic regression, and association rule learning network results. Venn diagram comparisons were constructed from our female seasonality analysis and our SAD regression results, risk factors were denoted by red text, and protective factors were denoted by silver text. (**a**) Genotypic factor comparison reveals risk factors exclusive to seasonality. (**b**) Clinical factor comparison reveals shared and exclusive risk factors between seasonality and SAD in females.

CRY2 and PER3C were risk factors exclusive to seasonality symptoms (Figure 6a). PER2 was protective for seasonality; CLOCK3111, PER3B, and ZBTB20 were protective for SAD symptoms; and CRY1 and PER3-VNTR were protective against both outcomes (Figure 6a). Age and anxiety were clinical risk factors for both outcome, while belonging to the upper middle class was specific to seasonality (Figure 6b).

### 3.5. Mediation Analyses Reveal Robust Mediation of Seasonality Relative to SAD Symptoms

Mediation analysis revealed that MEQ partially mediated the association of PER2-GG/PER3B-GG with seasonality in males (Table 2; t_153_ = 2.0, *p* = 0.046). In females, MEQ fully mediated PER2-GG (Table 2; t_153_ = −2.0, *p* = 0.048), while MSF partially mediated PER2-GG (Table 2, t_153_ = 2.13, *p* = 0.034). Also, MSF was a full mediator for CRY1-CC/PER2-AG (Table 2; t_153_ = −2.5, *p* = 0.015), and MEQ was a partial mediator for CRY1-CG/PER3-VNTR-5,5 (Table 2; t_153_ = −2.5, *p* = 0.014).

### 3.6. Mutual Information Analyses

**Figure 7:**
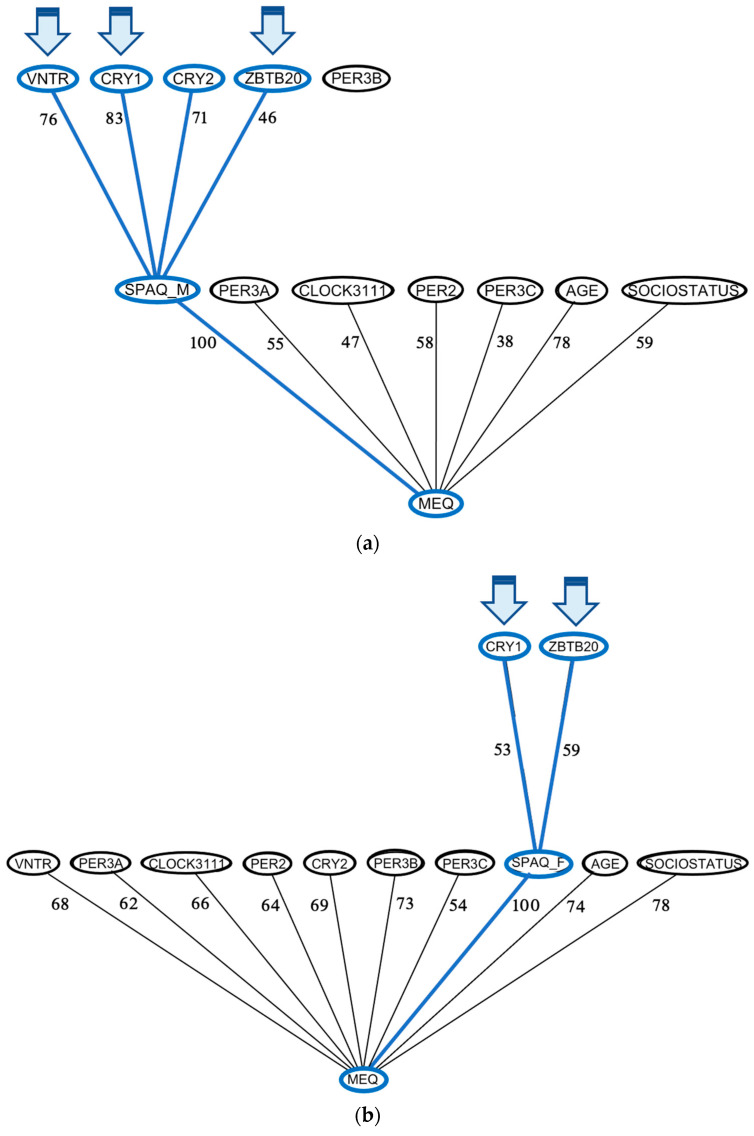
ARACNE networks for seasonality in male and female population subsections. ARACNE networks were constructed for seasonality in (**a**) males and (**b**) females using MEQ as a potential mediator variable.

**Figure 8:**
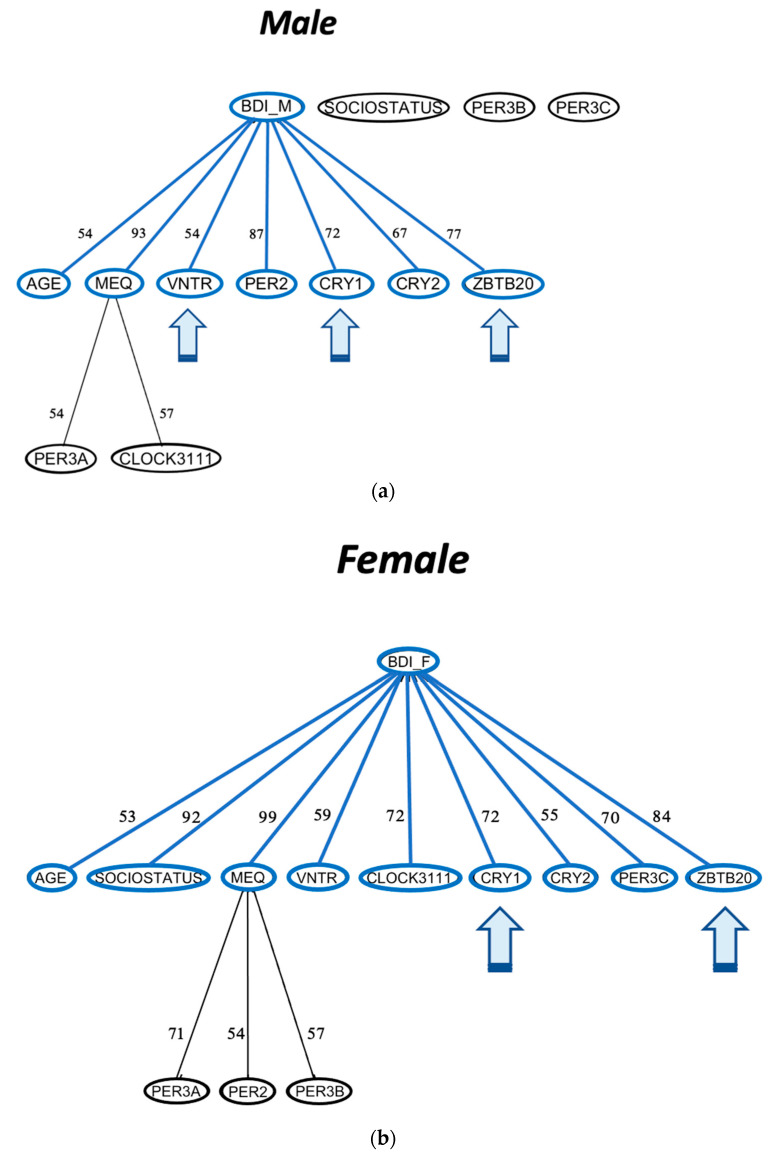
ARACNE networks for depression in (**a**) male and (**b**) female population subsections. ARACNE networks were constructed for depression in males and females using MEQ as a potential mediator variable.

ARACNE analysis revealed that CRY1 and ZBTB20 share mutual information directly with seasonality in females, while other genotypes were mediated through diurnal preference; males also show direct links between CRY1 and ZBTB20, as well as CRY2 and PER3-VNTR (Figure 7). When tested independently using depressive symptoms, CRY1, CRY2, PER3-VNTR, and ZBTB20 also had direct associations with depressive symptoms in both males and females (Figure 8).

## 4. Discussion

Although there is considerable evidence in the literature suggesting multiple connections between clock gene variants, circadian disruptions, and seasonal affective disorder, previous GWAS, PheWAS, and gene association studies have only been able to describe the effects of single clock gene mutations on seasonal affective disorder. These traditional techniques are limited in their ability to explore the effects of multiple genotype combinations or to distinguish between the direct or indirect effects of clock genes on SAD symptoms. Using novel machine learning approaches and statistical analyses, we observed effects of both single-genotype and multi-genotype combinations on SAD. Additionally, we were able to discern direct and indirect effects using ARACNE analyses combined with mediation analyses. We report three main findings from these analyses: (1) clock variants predictive of seasonal affective disorder tend to exhibit sex-specific effects; (2) clock variants can have direct transcriptional effects on seasonality and SAD symptoms or indirect effects that are mediated by diurnal preference or chronotype; and (3) clock variants with direct effects on seasonality symptoms also have direct effects on depressive symptoms.

### 4.1. Circadian Genotypes Exhibit Sex-Specific Effects on Seasonality and SAD Symptoms

Our feature selection results revealed greater prediction accuracy increases from the baseline when the dataset was stratified by sex. In addition, the main findings from our multiple logistic and linear regression analyses revealed sex-specific risk and protective factors. For example, PER2-GG/PER3B-AG and CLOCK3111-TC/ZBTB20-AT were significantly associated with the risk of seasonal symptoms in males, and CRY1-GG/PER2-AA, CRY2-AA/PER3C-TG, CRY2-AA/PER3-VNTR-4,5, PER2-AA/PER3C-TT, and PER3-VNTR-5,5/CRY1-GG had significant associations with the risk of seasonal symptoms in the female population. These findings suggest that these genotypic combinations of clock genes exert sex-specific effects, a pattern previously identified in studies examining major depressive disorder (MDD) [18] and anxiety [31].

Interestingly, Shi et al. (2016) found that the CLOCK variant (rs1801260) was significantly associated with MDD in males only, a pattern similar to our finding of a sex-specific association of a genotype combination (CLOCK3111-TC/ZBTB20-AT) with seasonal symptoms in males. This CLOCK variant may be involved in sex-specific effects through glucocorticoid regulation [18]. Also, since CLOCK3111-TC has been associated with the sleep–wake cycle [57,58], this polymorphism may promote circadian disruption, which is hypothesized to affect mood through serotonin regulation [59]. This mechanistic reasoning is in line with clinical evidence, which has demonstrated that SSRIs can eliminate SAD in affected individuals [60].

### 4.2. Clock Gene Effects on Seasonality and SAD May Be Direct or Mediated by Diurnal Preference

The relationship between seasonality score and chronotype is well-established [61,62,63], indicating that circadian gene variants associated with chronotype and circadian phase shifts may indirectly affect seasonal depressive symptoms through a lack of energy, burnout, sleeping problems, and social withdrawal, which may result from physiological misalignment of circadian rhythms with behavioral patterns [64]. In support of this, our current study found that most of the variables in the ARACNE analyses were mediated via diurnal preference (MEQ) and/or chronotype (MSF). For example, CRY2 and PER3C were risk factors exclusive to seasonality symptoms, and variants of these two genes have previously been associated with advanced and delayed sleep phase [65,66], respectively, suggesting that these genes are critical to circadian clock pathways. We also found that PER2, a protective factor exclusive to seasonality, was mediated by diurnal preference and appeared in several genotypic combinations that were also mediated by diurnal preference, further supporting the importance of sufficient PER2 gene function to maintaining circadian clock alignment.

Although the PER3-VNTR and CRY1-associated genotypes were protective for both seasonality and SAD analyses, mediation analysis found that the CRY1/PER3-VNTR protective combination was partially mediated through diurnal preference, raising the possibility that these genes may also be involved in pathways that indirectly influence mood. Indeed, the PER3-VNTR 4 allele has been associated with anxiety [67,68] and depression [69], in addition to evening chronotype [12,13]. Genotypes with variants of PER3B and CLOCK3111 were also protective for SAD in this population. In the Shi et al. (2016) study, the PER3B A allele (rs17031614) was significantly associated with depression, and the CLOCK3111 C allele was significantly associated with MDD in males [18], suggesting potential sex-specific, direct effects on mood. However, we observed in our ARACNE analyses that genotype combinations with the PER3B and CLOCK3111 genes were mediated by diurnal preference. Moreover, the CLOCK3111 C-allele has previously been associated with evening chronotype [58] and seasonal depression [14], suggesting indirect effects or a combination of direct and indirect effects for this gene.

On the other hand, our ARACNE analysis indicates that genotypes including variants of ZBTB20 and CRY1 may have robust direct effects on mood as these two genes that shared mutual information directly with seasonality and are not mediated by diurnal preference or chronotype. In other studies, CRY1 is robustly associated with depression [4,24]. The ZBTB20 gene is currently the only gene associated with SAD in GWAS studies [70] and directly shares mutual information with seasonality in our study. Together, these findings suggests that CRY1 and ZBTB20 are genes involved directly in mood regulation.

### 4.3. Clock Variants with Direct Effects on Seasonality Symptoms Also Have Direct Effects on Depression Symptoms

Seasonal affective disorder is classified as a subset of depression where a small portion of individuals exhibiting clinical depression may also exhibit seasonal variability characteristic of SAD. Clock gene associations are commonly consistent between those with SAD [17,71,72] and depression [4,15]. Given this overlap, we utilized patient depression scores (BDI) as the outcome for ARACNE analysis to test the hypothesis that PER3-VNTR, CRY1, and ZBTB20 are directly involved in mood regulation. Here, we observed that the effects of CRY1, PER3-VNTR, and ZBTB20 were also directly associated with depression, underscoring the possibility that these genes directly impact mood regulation via transcriptional (CRY1, PER3-VNTR) or activational (ZBTB20) regulatory mechanisms.

The PER3-VNTR genotype was directly associated with depression in both males and females, although it was only directly associated with seasonality in males only. This SNP (rs57875989) has previously been associated with anxiety and depression [24,68], but has also appeared significantly associated with evening chronotype [12,13], suggesting that PER3-VNTR may have both direct and indirect effects on mood disorders. Liberman et al. (2018) found that the PER3-VNTR 4-repeat allele was associated with both a three-fold increase in odds of depression and significantly greater tendencies toward eveningness in the same dataset [24]. Previously, Archer et al. (2003) identified an association between this polymorphism and extreme diurnal preference, finding that those homozygous for the 5-repeat allele are more likely to be morning-types, while those homozygous for the 4-repeat allele are more frequently evening types and encounter delayed sleep phase disorder (DSPS) [12]. The PER3-VNTR polymorphism occurs in exon 18, encoding a variable number of tandem repeat region (VNTR) of 18 amino acids that repeats four or five times depending upon the variant allele [73]. PER3-VNTR appears to have an important role regulating sleep homeostasis as this gene has been implicated in sleep duration, timing, and structure. These findings have led others to hypothesize that PER3-VNTR may function in a shared molecular pathway involving the regulation of circadian rhythm and sleep structure, although such a pathway has yet to be confirmed by experimental findings. Supporting our observed gender effect, Weiss et al. (2020) found that PER3^4,4^ males had nine times greater odds of being evening-types, while there was no significant effect in females or the combined dataset [73]. Such findings support the notion that PER3-VNTR may influence mood directly through sex-specific pathways illuminated above such as glucocorticoid or serotonin regulation.

Previous research illuminates the potential pathways by which CRY1 and ZBTB20 variants may directly affect seasonal affective disorder. The CRY1 SNP (rs2287161) is located upstream of the CRY1 promoter [4], and it has been predicted that this SNP fosters increases in CRY1 gene expression [24]. CRY1 has a critical role in HPA axis regulation via the repression of glucocorticoid receptors [74], and dysregulation of this pathway may promote the development of depressive symptoms [75]. Therefore, differential expression of CRY1 could affect glucocorticoid receptor repression and HPA axis stability, contributing to depressive symptoms. Altered expression of CRY1 also likely affects the expression of other circadian genes [24], which may then directly alter mood through physiological changes in other downstream mood pathways.

The ZBTB20 (rs139459337) SNP results in reduced expression of the ZBTB20 gene [22], which has been associated with seasonal affective disorder and major depressive disorder [22,76]. ZBTB20 is a zinc finger transcriptional repressor protein that is abundant in the hippocampus and plays an important role in the regulation of clock gene expression. Indeed, the loss of ZBTB20 is associated with impaired output of circadian rhythms [77,78]. As a transcription factor, ZBTB20 is also involved in synaptic transmissions, which could influence glucose homeostasis, glucocorticoid stress pathways, or monoamine pathways associated with depression symptoms. Our observations from the ARACNE analyses suggest direct effects of CRY1 and ZBTB20 on seasonal mood symptoms as well as depressive symptoms, supporting these previous findings of associations of CRY1 and ZBTB20 on MDD [4,24]. However, the precise mechanisms by which ZBTB20 and CRY1 exhibit direct effects on seasonal depressive symptoms are not yet known.

Our findings should be considered in light of the following limitations. First, our study population was relatively small and was composed predominantly of Caucasians of European descent, which limits the generalizability of our findings. Additionally, our analysis was constrained by the small prevalence of seasonal affective disorder in the male population, which prevented us from determining features important to SAD in males. Another limitation is that the current study focuses on the effects of chronotype, and we did not measure other aspects of circadian rhythmicity, like circadian amplitude or distinctness, that may intersect with seasonal affective disorder. Finally, seasonality in undergraduate students is potentially confounded by different sleep–wake schedule requirements in the summer months or with other unmeasured variables that differ by season.

## 5. Conclusions

In this study, we report the sex-dependent and direct and indirect effects of circadian genes on seasonal affective disorder. For example, several clock-related genes show sex-specific effects that may alter glucocorticoid and serotonin regulatory pathways. Our results also suggest that CRY1 and ZBTB20 are involved in pathways that directly influence mood symptoms involved in both SAD and MDD, while PER2, CRY2, PER3C, and PER3-VNTR variants show indirect effects via mediation by diurnal preference or chronotype. Understanding the physiological pathways directly or indirectly associated with SAD can lead to effective clinical targets or behavioral therapies, respectively, to help reduce the burden associated with SAD and other mood disorders.

## Figures and Tables

**Table 1:** Significant risk and protective factors for male seasonality, female seasonality, and female seasonal affective disorder (*p* < 0.05). Univariate and multivariate linear and logistic regression were performed for genotypic features in association with male and female seasonality (SPAQ M and F), while univariate and multivariate logistic regression were performed for female seasonal affective disorder (SAD F). Results from each separate analysis are grouped by outcome variable (SPAQ M, SPAQ F, SAD F), and univariate logistic odds ratios are enclosed in asterisks (risk factor: *odds ratio* > 1), multivariate linear estimates appear in brackets (risk factor: [estimate] > 0), and multivariate logistic odds ratios appear as plain numbers (risk factor: odds ratio > 1).

Feature	Male	Female	
Risk	SPAQ M OR [estimate]	SPAQ F OR [estimate]	SAD F OR [estimate]
CLOCK3111-TC/ZBTB20-AT	3.975		
CRY2-AA/PER3C-TG		[4.279]	
CRY2-AA/VNTR-4,5		*8.919*	
PER2-GG/PER3B-AG	7.262		
Anxiety			1.061
Evening type		*1.783*	
Increasing Age		*6.406*	
Sleep Disturbance	[0.320]	[0.173]	
**Protective**			
VNTR-4,5			0.258
CLOCK3111-TC/PER3B-GG			0.357
CLOCK3111-TC/PER3C-TT			0.383
CRY1-GG			0.491
CRY1-GG/PER2-AA		[−3.595]	
CRY1-GG/VNTR-5,5		*0.112*	
CRY1-GG/ZBTB20-AA			0.454
PER2-AA/PER3C-TT		*0.203*	
PER3B-GG/VNTR-4,5			0.304
PER3C-TT/VNTR-4,5			0.361
VNTR-4,5/ZBTB20-AA			0.321
Morning type	0.937		

**Table 2:** MEQ- and MSF-mediated associations of genotypic features with male and female seasonality. Mediation analysis was performed using the Horne-Östburg Morning–Eveningness Questionnaire (MEQ) and mid-sleep point on a free day [37] as potential mediators, and mediation was confirmed for average mediated effect *p*-values < 0.10. Mediation was considered full if the average direct effect had a *p*-value ≥ 0.10.

Genotypic Feature	SPAQ F	SPAQ M
PER2-GG	Full MEQ, partial MSF	
CRY1-CC/PER2-AG	Full MSF	
CRY1-CG/VNTR-55	Partial MEQ	
PER2-GG/PER3B-GG		Partial MEQ

## Data Availability

The data presented in this study are openly available at https://github.com/thanhdang2712/Mood-Disorder-Project.
